# Early microbial and metabolomic signatures predict later onset of necrotizing enterocolitis in preterm infants

**DOI:** 10.1186/2049-2618-1-13

**Published:** 2013-04-16

**Authors:** Ardythe L Morrow, Anne J Lagomarcino, Kurt R Schibler, Diana H Taft, Zhuoteng Yu, Bo Wang, Mekibib Altaye, Michael Wagner, Dirk Gevers, Doyle V Ward, Michael A Kennedy, Curtis Huttenhower, David S Newburg

**Affiliations:** 1 Perinatal Institute, Cincinnati Children’s Hospital Medical Center, Department Pediatrics, University of Cincinnati College of Medicine, 3333 Burnet Ave, MLC 7009, Cincinnati, OH 45229, USA; 2 Department of Environmental Health, University of Cincinnati College of Medicine, Cincinnati, OH, USA; 3 Department of Biology, Boston College, Chestnut Hill, MA, USA; 4 Department of Chemistry and Biochemistry, Miami University, Oxford, OH, USA; 5 Division of Biostatistics and Epidemiology, Cincinnati Children’s Hospital Medical Center, Department of Pediatrics, University of Cincinnati College of Medicine, Cincinnati, OH, USA; 6 Division of Biomedical Informatics, Cincinnati Children’s Hospital Medical Center, Department of Pediatrics, University of Cincinnati College of Medicine, Cincinnati, OH, USA; 7 Broad Institute, Cambridge, MA, USA; 8 Harvard School of Public Health, Boston, MA, USA

**Keywords:** Microbiome, Premature infants, Necrotizing enterocolitis, Dysbiosis

## Abstract

**Background:**

Necrotizing enterocolitis (NEC) is a devastating intestinal disease that afflicts 10% of extremely preterm infants. The contribution of early intestinal colonization to NEC onset is not understood, and predictive biomarkers to guide prevention are lacking. We analyzed banked stool and urine samples collected prior to disease onset from infants <29 weeks gestational age, including 11 infants who developed NEC and 21 matched controls who survived free of NEC. Stool bacterial communities were profiled by 16S rRNA gene sequencing. Urinary metabolomic profiles were assessed by NMR.

**Results:**

During postnatal days 4 to 9, samples from infants who later developed NEC tended towards lower alpha diversity (Chao1 index, *P* = 0.086) and lacked *Propionibacterium* (*P* = 0.009) compared to controls. Furthermore, NEC was preceded by distinct forms of dysbiosis. During days 4 to 9, samples from four NEC cases were dominated by members of the Firmicutes (median relative abundance >99% versus <17% in the remaining NEC and controls, *P* < 0.001). During postnatal days 10 to 16, samples from the remaining NEC cases were dominated by Proteobacteria, specifically Enterobacteriaceae (median relative abundance >99% versus 38% in the other NEC cases and 84% in controls, *P* = 0.01). NEC preceded by Firmicutes dysbiosis occurred earlier (onset, days 7 to 21) than NEC preceded by Proteobacteria dysbiosis (onset, days 19 to 39). All NEC cases lacked *Propionibacterium* and were preceded by either Firmicutes (≥98% relative abundance, days 4 to 9) or Proteobacteria (≥90% relative abundance, days 10 to 16) dysbiosis, while only 25% of controls had this phenotype (predictive value 88%, *P* = 0.001). Analysis of days 4 to 9 urine samples found no metabolites associated with all NEC cases, but alanine was positively associated with NEC cases that were preceded by Firmicutes dysbiosis (*P* < 0.001) and histidine was inversely associated with NEC cases preceded by Proteobacteria dysbiosis (*P* = 0.013). A high urinary alanine:histidine ratio was associated with microbial characteristics (*P* < 0.001) and provided good prediction of overall NEC (predictive value 78%, *P* = 0.007).

**Conclusions:**

Early dysbiosis is strongly involved in the pathobiology of NEC. These striking findings require validation in larger studies but indicate that early microbial and metabolomic signatures may provide highly predictive biomarkers of NEC.

## Background

Preterm birth contributes disproportionately to the global burden of morbidity and mortality in infancy. Necrotizing enterocolitis (NEC) is a devastating emergency of preterm infants, which affects about 10% of infants born <29 weeks gestational age, with a case-fatality of about 30% [1,2]. NEC typically occurs without clinical warning between 3 days and several months of postnatal life [3]. Risk factors for NEC include immaturity [4], timing and type of infant feeding [5,6], extended empirical use of antibiotics [7,8], and intestinal bacterial colonization [9-12]. Unfortunately, after decades of research, the specific pathogenesis of NEC remains an enigma, and validated biomarkers to identify individuals at highest risk of disease are lacking.

Intestinal colonization has remained an important target of investigation in the etiology of NEC. The microbial community of preterm infants, compared to healthy, term infants, consists of dramatically fewer beneficial species, lower bacterial diversity, and more pathogens [9-12]. NEC does not occur in germ-free animals, lending credence to the importance of intestinal colonization to the development of NEC [13]. Several epidemiologic studies in preterm infants report an association between early empirical antibiotic use and subsequently increased risk of NEC, while randomized, controlled trials in preterm infants suggest that probiotic agents may reduce the risk of NEC [7,8,14]. The immature enterocytes of preterm infants exhibit excessive signaling in the TLR4 pathway in response to the lipopolysaccharide (LPS) presented by Gram-negative bacteria, and this interaction has often been implicated in NEC onset [12,13,15]. Various other agents have also been associated with NEC onset, including bacterial, viral, and fungal pathogens [9-11,16-18]. Despite this, no specific microbial pattern has been consistently identified with NEC onset.

Early colonizing organisms interact with the intestinal mucosa to shape the developing immune system towards homeostasis or dysregulation [19-24], and could thus contribute to the pathobiology leading to onset of NEC. However, early colonization has been largely overlooked as a focus of study. To address this gap, we examined the early microbial community to identify predictive microbial biomarkers of later NEC in a prospective study of preterm infants. Culture-independent 16S rRNA gene sequencing of stool samples from 4 to 16 days of life was utilized to identify microbial community signatures. Because intestinal bacteria can influence the metabolic profiles of their hosts [25-29], we pursued urinary metabolomics to identify surrogate biomarkers of NEC.

## Methods

### Preterm infant cohort

All infants in this study were part of an ongoing larger study of the preterm microbiome and determinants of NEC, which was conducted in two level III neonatal intensive care units (NICUs) in Cincinnati, Ohio. Infants were enrolled between October 2009 and August 2010. Enrollment criteria included being free of congenital anomalies and survival free of NEC in the first week of postnatal life. After Institutional Review Board approval and parental informed consent, standardized clinical data were collected following the National Institute of Child Health and Human Development (NICHD) Neonatal Research Network protocol until discharge from hospital. All cases were reviewed by a senior neonatologist (KRS).

A total of 35 preterm infants <29 weeks gestational age and <1,200 g birth weight were included in this study. The primary analysis included 11 infants who developed NEC and 21 control infants. As a secondary comparator, we included three non-NEC deaths attributed to respiratory distress syndrome or suspected infection. Non-NEC deaths were included as they represent a competing outcome with NEC during hospitalization in the NICU, and clinical studies of probiotics and antibiotics report similar associations with NEC and death [7,8,14], suggesting the possibility that they may be similarly related to the intestinal microbiome. Controls were consecutively enrolled infants from the same NICUs who survived free of NEC or sepsis. NEC was defined as modified Bell’s stage II or III [30].

The infant microbiome was analyzed by postnatal time periods, days 4 to 9 and 10 to 16. These periods were selected because the extremely premature infants in this study did not stool consistently and did not stool prior to day 4 of life. Each period included two planned sample collections, increasing the chances of having a sample available from each infant during the interval. Stools were collected from soiled diapers; urine samples were collected from the infant diaper using cotton balls. Upon collection, samples were immediately refrigerated in the NICU and transported to the study laboratory daily. Stool was scraped into a tube containing thioglycollate, and frozen at −80°C until the DNA could be extracted. Upon removal from the cotton ball, urine samples were frozen at −80°C without an additive until metabolomic analysis was performed. All sample collection and storage utilized preprinted, bar-coded labels.

In the few instances when two stool samples were available per infant in a collection period, statistical independence was ensured by including only the earlier sample. Further, only samples collected prior to diagnosis of NEC were included. These criteria resulted in inclusion of 58 stool samples for analysis, 18 from the 11 NEC cases, 37 from the 21 control infants, and 3 from the 3 non-NEC deaths. Urine samples were collected during days 4 to 9, and all available samples collected prior to NEC onset in that time period were analyzed by NMR [31]. A total of 60 urine samples, 18 from 11 NEC cases, 36 from 20 controls, and 6 from non-NEC deaths, were included in initial analyses. Final analysis of urinary biomarkers was restricted to the first urine sample collected in the day 4 to 9 period.

### DNA extraction

Bacterial DNA was extracted from infant stool samples using one of two methods: phenol-chloroform or the QiaAmp DNA stool kit (Qiagen Sciences, Germantown, MD). For the phenol-chloroform method, samples were thawed, centrifuged and supernatant removed. From the sample, 0.2 g of stool was transferred to a 2 mL screw-cap tube containing 0.3 g of 1 mm zirconia beads. Next, 1 mL Tris-EDTA (TE) buffer was added and the sample resuspended. Then, 150 μL of buffer saturated phenol was added and the sample bead beat for 3 minutes at 4°C. The sample was then allowed to sit for 1 minute at 4°C before extraction with 150 μL chloroform:isoamyl alcohol (24:1). Samples were centrifuged for 5 minutes at 15,700*g* at 4°C and the resulting aqueous layer was transferred to a clean tube and again extracted with an additional 150 μL phenol and 150 μL chloroform:isoamyl alcohol. Samples were then centrifuged for 2 minutes at 15,700*g* at 4°C. The aqueous layer was subjected to two additional chloroform:isoamyl alcohol extractions. The resulting aqueous layer was transferred to a clean microfuge tube a final time and 4 μL of 5 mg/mL glycogen was added and mixed. This was followed by the addition of 1/10 volume of 3 molar sodium acetate and 2 volumes of 70% cold (−20°C) ethanol. The samples were mixed and then centrifuged at 15,700 *g* for 5 minutes at 4°C. The supernatant was discarded and the pellet resuspended in 100 μL TE buffer. For the extractions using the QiaAmp DNA stool kit, samples were thawed and 0.2 g of stool was transferred to a bead beating tube containing 0.3 g of 0.1 mm glass beads, 1.4 mL of buffer ASL was added, and bead beating was conducted for 3 minutes on the homogenize setting. The suspension was then heated at 70°C for 5 minutes, and the manufacturer’s instructions followed.

### 16S rRNA gene analysis

The V3 to V5 window of the 16S rRNA gene was amplified and sequenced using 454 FLX Titanium sequencing as already described in full detail [32]. A total of 1.3 million resulting sequences were processed using a data curation pipeline implemented in mothur [33], complemented by UCHIME for chimera detection [34]. This processing has been detailed previously [35], with the modification that AbundantOTU was replaced by Newbler for assembly-based error reduction [36] and followed by mothur's implementation for operational taxonomic unit (OTU) clustering (parameters: method = average, cutoff = 0.03, precision = 1,000). The mean read count per sample was 4,989. Representative sequences per OTU were classified with the MSU RDP classifier v2.2 [37] using the Greengenes taxonomy [38]. The 16S rRNA gene sequences obtained from the study samples were assigned to a total of 411 distinct OTUs. OTUs were then assigned phylogenetic classifications, typically to the genus level.

### Metabolomic analysis

Urine samples were thawed on ice immediately prior to preparation for NMR analysis. A 1 mL aliquot of each sample was centrifuged for 10 minutes at 2,655 *g*, 350 µL of clarified urine pipetted into a 1.5 mL microcentrifuge tube, and 350 µL of buffer added to each sample. Next 600 µL of the urine/buffer mixture was pipetted into a 5-mm NMR tube. All NMR experiments were carried out on a Bruker Avance™ III spectrometer operating at 600 MHz ^1^H frequency equipped with a room-temperature 5-mm triple-resonance probe with inverse detection and controlled by TopSpin 3.0 (Bruker Biospin, Germany). Experiments were conducted at 298 K. Data were collected using a spectral width of 20.0 ppm. Three ^1^H NMR experiments, optimized by Bruker (Bruker BioSpin, Germany) for use in metabonomic studies, were run on all samples: a standard one-dimensional (1D) presaturation (zgpr), a 1D first increment of a NOESY (noesygppr1d), and a CPMG (cpmgpr1d); however, only the CPMG, which produced superior baselines for analysis, was used for metabolomic analysis. The transmitter offset frequency (O1) was adjusted to obtain optimal water suppression. The 90° pulse widths, determined for every sample using the automatic pulse calculation feature in TopSpin, were between 10 and 12 μs. Water suppression in all experiments was achieved by irradiation of the water peak during the recycle delay. Next 1D zgpr ^1^H NMR spectra were collected to assess the shim quality, which was considered acceptable when the line width was <1 Hz and the line shape enabled detection of resolved C13 satellites for the TSP internal standard. The CPMG experiment used 64 transients with 4 dummy scans, 46,280 points per spectrum giving an acquisition time of 1.87 s, a T_2_ filter loop of 128 with an echo time of 1 ms, apodized using −0.01 Hz of exponential line broadening, and a 4-s recycle delay. All NMR spectra were phased, baseline corrected, and subjected to chemical shift registration relative to TSP in TopSpin 3.0 (Bruker BioSpin, Germany).

### Statistical methods

Differences in clinical characteristics of cases and controls were tested using Fisher’s exact test for categorical variables and analysis of variance, *t*-tests or Kruskal–Wallis for continuous variables, as appropriate. The Kruskal–Wallis test was used to compare the relative abundance of distinct taxonomic units. Data were analyzed using R [39], linear discriminant analysis effect size (LEfSe) [40], QIIME [41,42], and SAS [43]. LEfSe was used to identify the phylogenetic features that differed significantly between all NEC cases and controls and later between NEC sub-types and controls; this program uses Kruskal–Wallis tests to identify differences in abundance between groups at the alpha level of 0.05 [40].

### Microbial community analysis

We first examined alpha diversity [44,45], which was analyzed using the Simpson diversity index and Chao1 richness index. Samples collected prior to NEC onset were compared to controls without elimination of rare reads but after rarefying (standardizing) to 2,000 sequence reads per sample.

To examine the beta diversity of microbial communities, we used non-metric dimensional scaling (NMDS) [46,47], undertaken in R, to ordinate the microbial communities of samples based on the weighted UniFrac distance calculated by QIIME. To improve signal to noise and reduce random error, we excluded rare OTUs from the beta diversity analyses. Rare OTUs were defined as those detected in only one sample or with less than five sequences in the overall dataset [10]. This resulted in 99 distinct OTUs that were included for analysis.

Ordination using NMDS was undertaken with several random starts to avoid entrapment in local optima. The results were centered and scaled, so that the variance was maximized along the first NMDS axis. This method of ordination was chosen because NMDS measures the closeness of fit (stress) based on ranking of the dissimilarity of values, with no assumption of multivariate normality, and is a powerful and flexible method that handles sparse, non-parametric data well. Use of the weighted UniFrac distance metric was selected, as it accounts for the relative abundance and relatedness of taxa, rather than merely their presence or absence. Selection criteria for the number of dimensions in NMDS analysis were based on the protocol used in PC-ORD 6 software [47]. For each ordination, we then ran a cluster analysis on the UniFrac distance matrix using the Ward minimum variance method [48] to objectively identify clusters of samples with similar microbial composition. In this method, the distance between two clusters is the analysis of variance sum of squares between the clusters summed over all the variables. At each step, the within-cluster sum of squares is minimized over all partitions obtainable by merging clusters from the previous generation in order to maximize the likelihood at each level of the hierarchy. The number of clusters is determined by examining the scree plot resulting from the pseudo F and T plots. We used the SAS PROC Cluster procedure to conduct the clustering analysis.

Metabolomics allows identification of distinct patterns of small molecules generated during both host and microbial cellular metabolism [25-29]. Urinary metabolomics was thus undertaken in search of surrogate biomarkers of dysbiosis and additional clues regarding the microbially distinct NEC cases in relation to controls and to each other. Urinary metabolite data were analyzed using principal component analysis (PCA) as implemented in AMIX (Bruker Biospin, Billerica, MA). NMR spectra were prepared for PCA using manual binning to avoid splitting of peaks. Individual metabolites were compared between NEC cases and controls using a *t*-test as implemented by Proc MULTTEST in the SAS 9.2 software (SAS Institute, Cary, NC). The Benjamini and Hochberg procedure [49] was used to control for multiple comparisons, using an adjusted *P* value of <0.05 [49]. After identification of significant metabolites, generalized estimating equations were applied to adjust for multiple samples per subject and to control for potential confounding factors. Only metabolites that were significant at <0.05 in both analyses were considered significant. A total of eight samples analyzed by NMR were excluded based on poor sample quality or as outliers based on the Hotelling T2 method [50].

## Results

### Preterm infant cohort

The characteristics of NEC cases versus controls are shown in Table 1. Controls were generally well matched to NEC cases on clinical factors, and did not differ in regard to birth weight (median 850 g), gestational age (median 26 weeks), race (37% black), gender (51% female), mode of delivery (66% Cesarean section), maternal antibiotic use at the time of delivery (51%), or infant antibiotic use for ≥5 days in the first week (34%). All study infants were fed the mother’s own milk or human donor milk; the timing and degree of feeding was not significant in relation to NEC. Only primiparity differed between NEC cases (64%) and controls (24%, *P* = 0.05), but was not significantly associated with microbial composition. Of the 11 NEC cases, 8 were Bell’s stage II and 3 were Bell’s stage III (surgically treated). Four of the NEC cases died, and three of the NEC cases developed sepsis (two *Klebsiella* isolates, one coagulase-negative *Staphylococcus* isolate). The three non-NEC deaths included for comparison were white, male, and younger (24 and 25 weeks gestational age) and smaller (<850 g birth weight) than the other infants, but otherwise unremarkable. For access to the metadata, please see Additional file 1.

**Table 1:** Characteristics of study infants

**Variable, description**	**NEC**	**Controls**
	***n* = 11**	***n *** **= 21**
Birth weight, mean (SD) (g)	791 (212)	839 (187)
Gestational age, mean (SD) (weeks)	25.5 (1.8)	25.9 (1.5)
Non-Hispanic black, number (%)	5 (45.5)	8 (38.1)
Male, number (%)	6 (54.6)	8 (38.1)
Patent ductus arteriosus (PDA), number (%)	7 (63.36)	12 (57.1)
Primiparous, number (%)	7 (63.6)^a^	5 (23.8)
Prenatal steroids given (any), number (%)	10 (90.9)	19 (90.5)
Hypertension/eclampsia, number (%)	2 (18.2)	7 (33.3)
Antepartum hemorrhage, number (%)	1 (9.1)	5 (23.8)
Multiple births, number (%)	1 (9.1)	7 (33.3)
Antepartum antibiotic therapy, number (%)	6 (54.6)	10 (47.6)
Cesarean section delivery, number (%)	7 (63.6)	14 (66.7)
Chorioamnionitis, number (%)	1 (9.1)	1 (4.8)
Antibiotic use in the first week for ≥5 d, number (%)	4 (36.4)	6 (28.6)

^a^NEC vs controls, primiparity *P* = 0.05.

NEC: necrotizing enterocolitis.

### Dominant organisms

Consistent with previous studies in preterm infants [12,51-53], the dominant phyla were Proteobacteria and Firmicutes, with a minor contribution (1% to 2%) from Bacteroidetes and Actinobacteria. The most common genera were, in order of relative abundance: *Enterobacter*, *Staphylococcus*, *Escherichia*, *Enterococcus*, *Leuconostoc*, *Lactococcus*, *Streptococcus*, and *Clostridia*. The first four genera accounted for more than 90% of the microbial sequence reads. *Pseudomonas*, another Proteobacteria often associated with NEC or sepsis in preterm infants, occurred in 31% of samples, while together Lactobacillaceae and Bifidobacteriaceae, beneficial Gram-positive organisms (of the phyla Firmicutes and Actinobacteria, respectively), were present in only 19% of samples.

**Figure 1:**
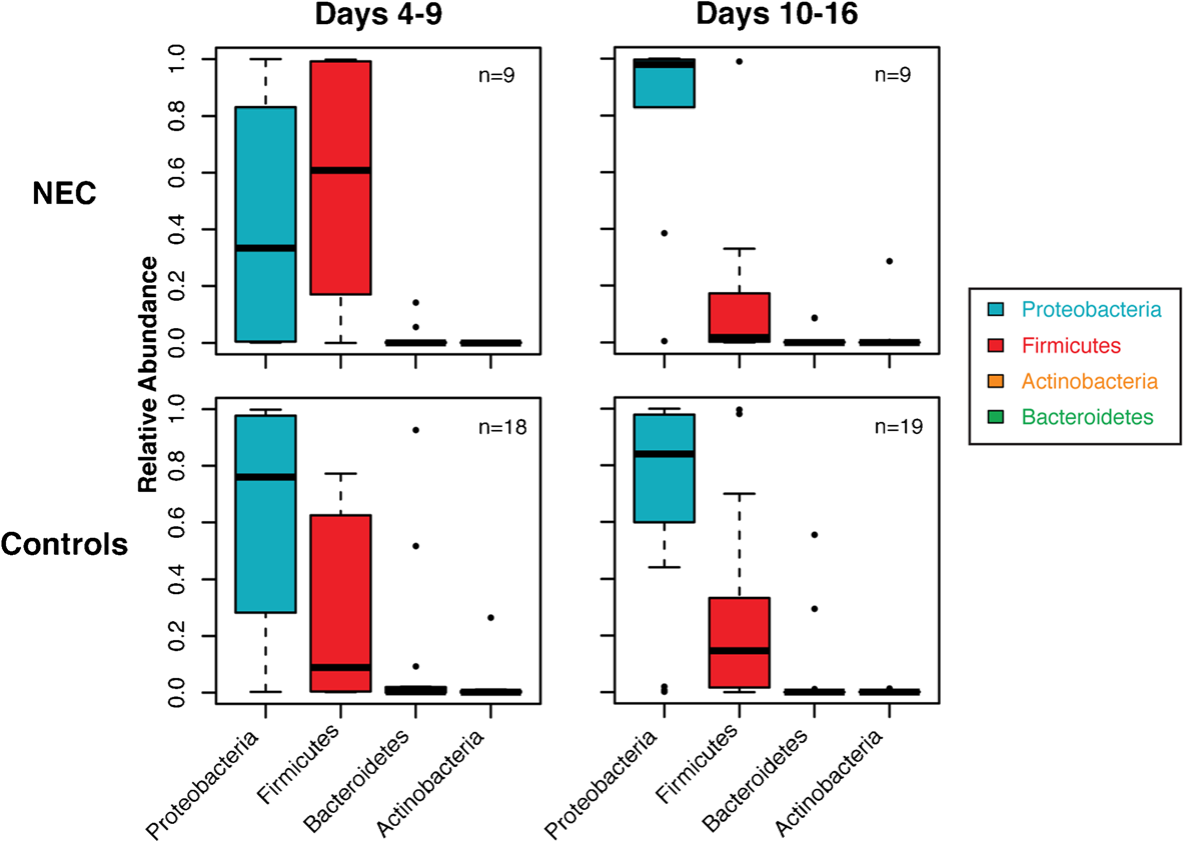
**The relative abundance of bacterial phyla in infants who developed NEC vs control infants.** Columns represent samples from days of life for 4 to 9 and 10 to 16. Data are graphed as box plots. The middle bar represents the median, the outer horizontal lines of the box represent the 25th and 75th percentiles. Dots above or below each box indicate outliers. NEC: necrotizing enterocolitis.

At the level of phyla, controls had a median relative abundance of approximately 80% Proteobacteria (Gram-negative organisms) and 20% Firmicutes (Gram-positive organisms), with a small proportion of Bacteroidetes and Actinobacteria; this pattern was remarkably stable over the first few weeks of life. Furthermore, most of the sequences contributing to the relative abundance of these large phyla came from only a few host-associated genera, *Enterococcus* and *Staphylococcus* for Firmicutes and *Enterobacter* and *Escherichia* for Proteobacteria. In infants who later developed NEC, microbial community composition differed sharply with the median relative abundance of Proteobacteria less than 40% and Firmicutes approximately 60% during postnatal days 4 to 9. A dramatic difference was then observed in samples from NEC cases such that over 90% of the microbial communities were composed of Proteobacteria, while less than 10% were composed of Firmicutes (Figure 1). In the three non-NEC deaths (data not shown), Firmicutes strongly dominated, accounting for 70% to 90% of microbial communities, with most of the remaining community composition being Proteobacteria.

**Figure 2:**
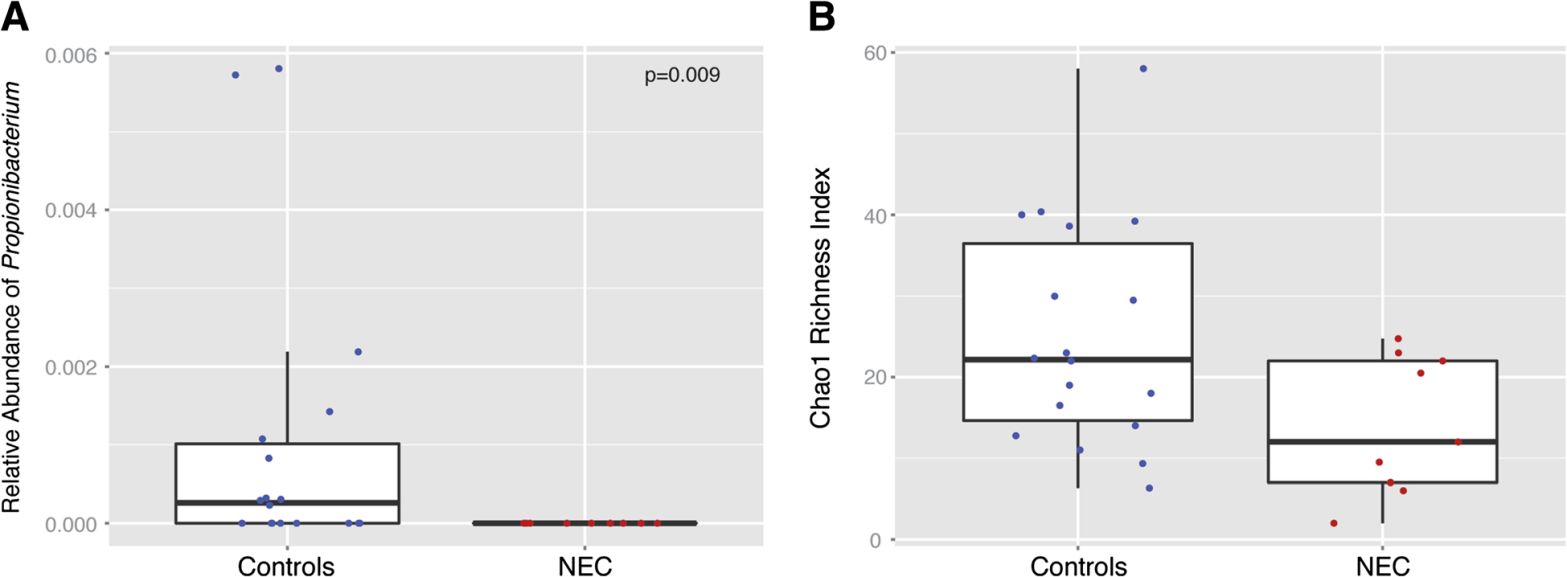
**Microbial community differences between NEC and control infants, days 4 to 9 samples.** (**A**) This box plot indicates the relative abundance of the genus *Propionibacterium* in 18 control samples and 9 samples prior to NEC onset during days 4 to 9 of life. None of the infants who later developed NEC, but 10 (56%) of the control samples had detectable amounts of *Propionibacterium* (using Fisher’s exact test for the presence or absence in NEC vs control samples, *P* = 0.009). (**B**) This box plot indicates the Chao1 richness index in samples from days 4 to 9 of life. NEC cases tended towards lower diversity than controls, but the comparison was not significant (Kruskal–Wallis, *P* = 0.086). For each box plot, the middle bar represents the median, the outer horizontal lines of the box represent the 25th and 75th percentiles. The dots overlaying the plots indicate the values of individual samples. Dots are distributed horizontally in order to prevent overlapping. NEC: necrotizing enterocolitis.

Systematic comparison of all NEC samples and all control samples collected between postnatal days 4 to 16 found no significant differences in microbial composition. NEC and control samples were then compared by week, and the only significant difference occurred in the relative abundance of *Propionibacterium*, a genus of the phylum Actinobacteria. *Propionibacterium* includes both skin- and intestinal-tract-colonizing organisms; members have demonstrated probiotic as well as pathogenic potential [54,55]. During days 4 to 9, *Propionibacterium* was identified in samples from 10 (56%) of the 18 control infants, but none of the 9 infants who later developed NEC (*P* = 0.01, Figure 2A). No difference was found in the relative abundance of *Propionibacterium* between NEC and control samples collected from days 10 to 16 of life.

### Alpha diversity

During postnatal days 4 to 9, infants who later developed NEC tended to have samples with lower alpha diversity than controls as measured by the Chao1 index (median 9.2 for NEC, 18.4 for control samples; Kruskal–Wallis, *P* = 0.086; Figure 2B) with a similar trend using the Simpson index (*P* = 0.221, data not shown). After day 9 of life, NEC samples continued to trend towards lower median alpha diversity than controls, but no significant differences were found by either index.

### Dysbiosis-identified sub-types of NEC

**Figure 3:**
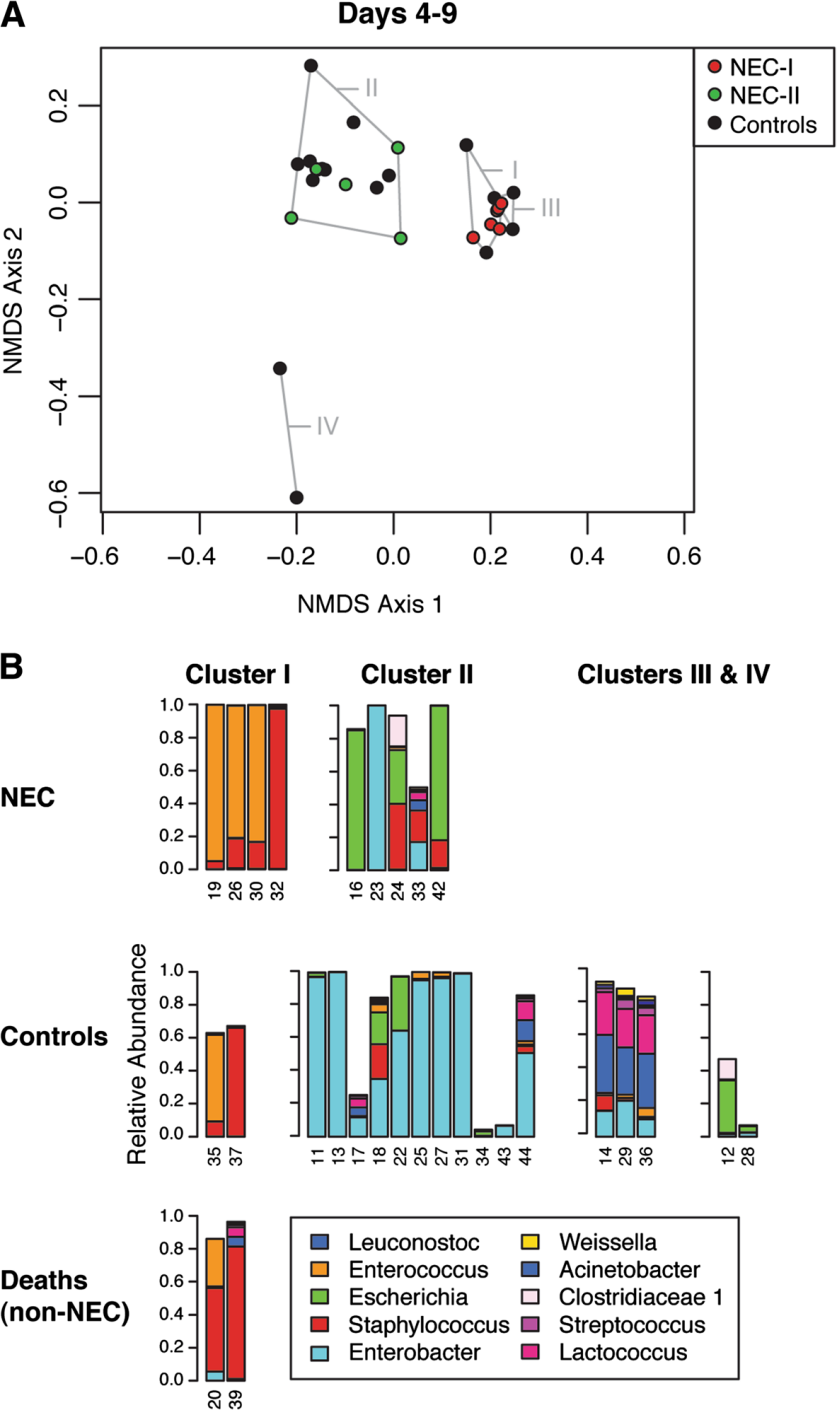
**NMDS ordination of microbial communities for days 4 to 9 of life.** (**A**) This ordination was based on weighted UniFrac beta diversity and run with three dimensions in the vegan package of R, resulting in a stress of 4.06. Control samples are shown in black and NEC and non-NEC deaths are shown in red for those included in Cluster I or green for those samples included in Cluster II. Clusters of samples with similar microbial composition were systematically identified using the Ward minimum variance method. These clusters are labeled using roman numerals I through IV. All NEC cases were found in either Cluster I or Cluster II only. The samples from the two non-NEC deaths are also found in Cluster I. (**B**) Bars indicate the relative abundance of the ten most common genera in samples from individual infants, whose study numbers are noted on the horizontal axis. Samples are grouped according to case or control status and the cluster in which they were identified. NEC: necrotizing enterocolitis; NMDS: non-metric dimensional scaling.

For all study infants, the relative similarity of microbial communities between samples (beta diversity) was then examined by calculating their weighted UniFrac distances, and visualized using non-metric multi-dimensional scaling ordination (NMDS). Based on ordination of day 4 to 9 samples (Figure 3A), application of the Ward minimum variance identified four clusters, designated I though IV, indicating microbial community similarity. Of these, only Clusters I and II included NEC cases. Cluster I consisted of samples from four NEC cases (NEC-I), two non-NEC deaths, and two controls (Figure 3B), and was characterized by dominance of organisms of the phylum Firmicutes, class Bacilli. Cluster II samples consisted of the remaining 5 NEC cases (NEC-II) as well as 12 of the 18 controls, and were characterized by dominance of organisms of the phylum Proteobacteria, family Enterobacteriaceae.

Within Cluster I, the genera *Enterococcus* and *Staphylococcus*, taxa representing different orders of Bacilli, accounted for 98% or more of the microbial community of samples from NEC-I infants. In the two non-NEC deaths found in Cluster I, the same taxa constituted 80% and 95% of their microbial communities, and in the two control samples, 62% and 73%. Comparing the NEC cases between clusters, the relative abundance of Firmicutes (specifically, Bacilli) was significantly higher in samples from NEC-I than NEC-II cases (median 99.3%, NEC-I versus 17%, NEC-II, *P* = 0.014). Further, NEC-I samples had a significantly greater relative abundance of Firmicutes, specifically, Bacilli (*P* = 0.001), compared to all controls. Conversely, the relative abundance of Proteobacteria, family Enterobacteriaceae, was significantly higher in samples from NEC-II than NEC-I cases (median 83%, NEC-II versus 0.4%, NEC-I, *P* = 0.014), but NEC-II samples did not differ significantly in microbial composition compared to all controls during days 4 to 9.

**Figure 4:**
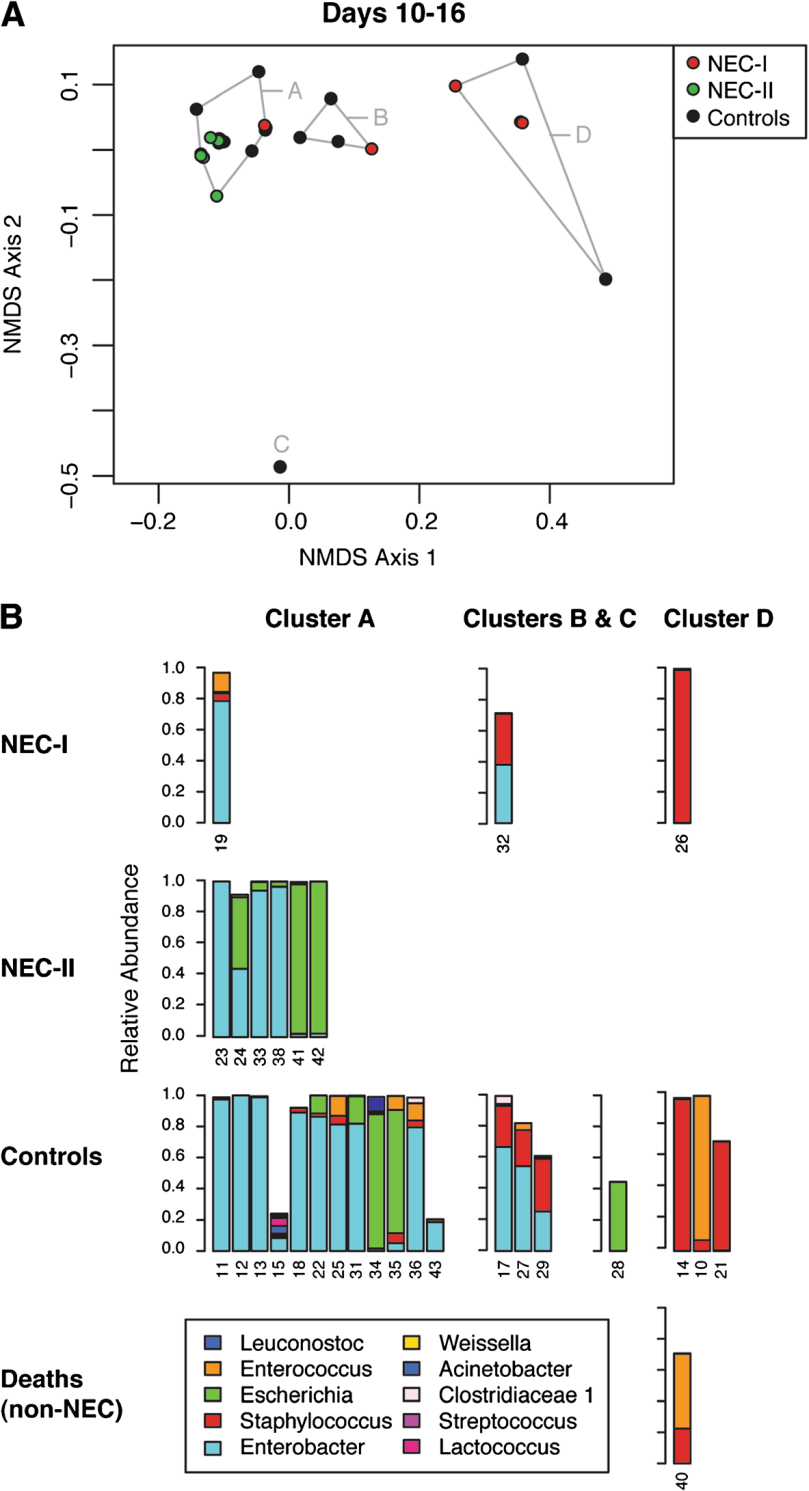
**NMDS ordination of microbial communities for days 10 to 16 of life.** (**A**) This ordination was based on weighted UniFrac beta diversity and run with three dimensions in the vegan package of R, resulting in a stress of 2.43. Controls are shown in black and NEC and non-NEC deaths in either red (cases identified as NEC-I in the ordination for days 4 to 9) or green (cases identified as NEC-II in the ordination for days 4 to 9). Clusters identified using the Ward minimum variance method are indicated in this ordination as A, B and D; C is identified as an outlier value. (**B**) Bars indicate the relative abundance of the ten most common genera in samples from individual infants, whose study numbers are noted on the horizontal axis. NEC sub-types (NEC-I and NEC-II) correspond to the NEC cases included in Cluster I and Cluster II, respectively, in the ordination of samples for days 4 to 9 (Figure 3). The clusters identified in this ordination are indicated by column headers. Clusters indicate microbial community similarity. NEC: necrotizing enterocolitis; NMDS: non-metric dimensional scaling.

The ordination of samples from days 10 to 16 (Figure 4A) and application of the Ward minimum variance method identified three microbial community clusters (A, B and D) and an outlier (C). The NEC-I infants that had tightly clustered in samples from the first 4 to 9 days were dispersed across Clusters A, B and D in this ordination, with no discernible similarity. In contrast, consistent with the ordination for days 4 to 9, all of the NEC-II cases were found in a single cluster (Cluster A) during days 10 to 16. Cluster A also included one of the dispersed NEC-I cases, the composition of which was 83% *Enterobacter*. Cluster A thus included 7 (78%) of the 9 NEC cases and 12 (63%) of 19 controls, and was characterized by preponderance of Proteobacteria, specifically, the family Enterobacteriaceae (Figure 4B). Compared to all control samples during days 10 to 16, the six NEC-II/Cluster A cases had a significantly elevated relative abundance of Proteobacteria (specifically, Enterobacteriaceae, *P* = 0.010). All six NEC-II/Cluster A infants versus 8 (42%) of 19 control samples were composed of 90% or more Proteobacteria (*P* = 0.020). We systematically compared the composition of samples, and found, as in the prior week, that during days 10 to 16, NEC-I samples had a significantly higher relative abundance of Firmicutes than NEC-II samples, specifically, Bacilli (median 33%, NEC-I versus 0.3%, NEC-II, *P* = 0.020). However, the relative abundances of these taxa in NEC-I cases during days 10 to 16 (median 33% Firmicutes or Bacilli) were not as extreme as that observed earlier (median ≥98% Firmicutes or Bacilli during days 4 to 9). In contrast, NEC-II cases became even more dominated by Proteobacteria, specifically, the Enterobacteriaceae (median 99.6%, NEC-II versus 38%, NEC-I, *P* = 0.020). Furthermore, during days 10 to 16, NEC-II samples also had a significantly greater relative abundance of Proteobacteria compared to all controls (median 99.6%, NEC-II versus 84%, controls, *P* = 0.01).

In summary, four NEC cases were classified as NEC-I, all of whom were in Cluster I, uniquely characterized by ≥98% relative abundance of Firmicutes, class Bacilli, during postnatal days 4 to 9. Six NEC cases were classified as NEC-II, all of whom were in Cluster A during days 10 to 16 as well as Cluster II during days 4 to 9. These NEC-II cases were all composed of ≥90% Proteobacteria, family Enterobacteriaceae, in samples from postnatal days 10 to 16. One NEC infant (subject 16) lacked a sample from days 10 to 16 and could not be formally classified, but followed the pattern of NEC-II based on their sample for days 4 to 9, which was dominated by *Escherichia* and is within Cluster II. All three non-NEC deaths were characterized by early Firmicutes dysbiosis, as for NEC-I. One of these non-NEC deaths (subject 40) lacked a sample from days 4 to 9, but was considered to be a high Firmicutes dysbiosis based on their sample for days 10 to 16, which was predominantly composed of *Staphylococcus*. The primary ordinations of beta diversity (Figures 3 and 4) included all samples and all non-rare sequence reads, but ordination using rarefied samples (Additional file 2: Figure S1) was also conducted and had the same pattern as that shown in Figures 3 and 4.

The alpha diversity of the microbial communities was then reanalyzed in relation to these NEC sub-types, as before using the full set of OTUs without eliminating rare sequence reads but rarefying samples to 2,000 sequence reads per sample. No significant differences were found in NEC sub-types by either the Simpson or Chao1 indices. We then compared the clinical characteristics of the NEC-I and NEC-II infants. NEC-I onset occurred between postnatal days 7 and 21, while NEC-II onset occurred between postnatal days 19 and 39 (*P* = 0.086, Kruskal–Wallis test). It is noteworthy that the non-NEC deaths, which clustered with NEC-I samples in the ordination of samples from days 4 to 9, had a similarly high Firmicutes dysbiosis in the first week of life, and that these deaths occurred between days 9 to 17, the same postnatal period during which NEC-I cases occurred. We then compared the two NEC sub-types in relation to each variable listed in Table 1, as shown in Additional file 2: Table S1. The only statistical difference in the two NEC sub-types was in the administration of antibiotics to the mother at the time of delivery. No NEC-I infants, but 6 (86%) of the 7 NEC-II infants, were born of mothers who were given antibiotics at the time of delivery (*P* = 0.015). While different delivery modes did not readily explain the association between NEC-II and maternal antibiotic use, we examined the beta diversity of the microbial communities in relation to delivery mode and NEC or control status, and observed a tendency for NEC-I to cluster with C-section delivery, and NEC-II to cluster with vaginal delivery (Additional file 2: Figure S2). For two other clinical factors, patent ductus arteriosus (PDA, a heart problem of preterm infants, which has been linked to later-onset NEC) and primiparity, we observed a trend (*P* = 0.089) towards a different distribution in NEC-I and NEC-II cases. Each factor was independently found in only 1 (25%) of 4 NEC-I infants versus 6 (86%) of 7 NEC-II infants. No other clinical characteristics appeared to differ between the NEC sub-types, nor in comparison to controls.

### DNA extraction

As DNA extraction methods can affect the results of studies of the 16S rRNA gene, we undertook a series of analyses to identify potential effects in our data. Examination of the extraction protocol in relation to beta diversity found no influence on identification of community clusters (Additional file 2: Figure S3A). Examination of the extraction protocol in relation to the relative abundance of bacteria at all taxonomic levels found taxa that differed by extraction method, but none of the taxa identified also differed significantly between NEC or NEC sub-types and controls (Additional file 2: Figure S3B). Finally, examination of the extraction protocol in relation to alpha diversity found no association with the Simpson index, but did find association with the Chao1 index (*P* = 0.01). Finally, we examined the extraction protocol in regression models of alpha diversity in relation to NEC, and found that it did not influence the findings. These data strengthen our conclusion that early differences occur in the microbiome of premature infants prior to onset of NEC, independent of extraction techniques.

### Urinary metabolomic analysis

**Figure 5:**
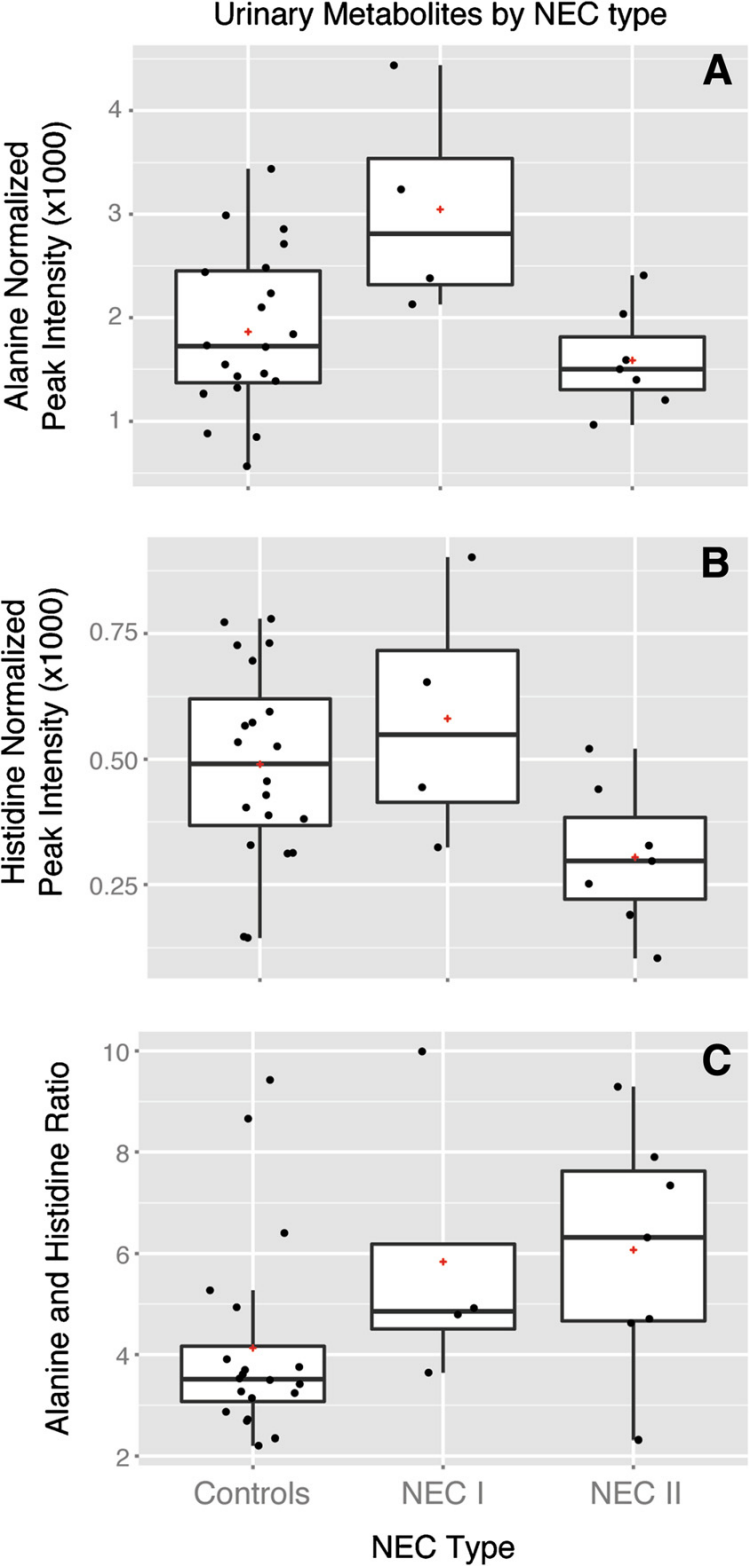
**Box plots of urinary alanine and histidine in relation to NEC sub**-**types versus controls.** Urine samples collected during days 4 to 9, restricted to one sample per infant (31 samples). Analysis of urinary alanine, pyridoxine and histidine among NEC-I, NEC-II, death and control subjects using multiple samples per individual (60 samples) is shown in Supplementary Information, Table S2 of Additional file 2. (**A**) Urinary alanine. Using the Kruskal–Wallis test, urinary alanine was significantly higher in NEC-I vs controls (*P* = 0.044) and NEC-I than NEC-II (*P* = 0.023), but did not differ between NEC-II vs controls or all NEC vs controls. (**B**) Urinary histidine. Using the Kruskal–Wallis test, urinary histidine was significantly lower in NEC-II vs controls (*P* = 0.023). Histidine also tended to be lower in NEC-II vs NEC-I (*P* = 0.059), but did not differ between NEC-I vs controls or all NEC vs controls. (**C**) Ratio of urinary alanine to histidine. Using the Kruskal–Wallis test, the ratio of urinary alanine to histidine was significantly higher in NEC cases overall vs controls (*P* = 0.023), but did not differ between the NEC sub-types. The optimal cut-point in the alanine:histidine ratio to differentiate NEC cases from controls was identified as a ratio greater than 4 (predictive value 78%, *P* = 0.007). NEC: necrotizing enterocolitis.

Principal component analysis did not demonstrate any qualitative clustering in the set of urinary metabolites in relation to all NEC cases, NEC-I or NEC-II versus controls, nor the NEC sub-types in relation to each other. Individual urinary metabolite values were compared using *t*-tests corrected for multiple comparisons. No urinary metabolites differed significantly among all NEC cases and controls. However, three metabolites, alanine, pyridoxine (4-pyridoxate) and histidine, significantly distinguished NEC-I and NEC-II from each other as well as one of the NEC sub-types from controls (Figure 5 and Additional file 2: Table S2). Alanine was significantly (*P* < 0.001) higher in NEC-I versus NEC-II and NEC-I versus the control samples though the metabolite did not differ between all NEC versus control samples (Figure 5A). Pyridoxine followed a pattern similar to alanine, though not as significant. The remaining metabolite, histidine (Figure 5B), differed in pattern and was significantly lower in NEC-II samples than controls (*P* = 0.01) and NEC-I samples (*P* < 0.001).

Alanine, pyridoxine and histidine are commonly synthesized by bacterial enzymes, as documented by KEGG [56] and may be considered plausible biomarkers of bacterial dysbiosis. We analyzed the relationship of these urinary metabolites to microbial community characteristics in our dataset, using only the first collected urine sample within the window for days 4 to 9 when multiple samples were available. Alanine (Table 2) was significantly associated with characteristics of the intestinal microbial community. Alanine levels were most strongly associated (*P* < 0.001) with Cluster I samples identified in the ordination for days 4 to 9, including the NEC-I cases, non-NEC deaths and the controls in that cluster. Alanine was also directly correlated with the relative abundance of Firmicutes (*P* = 0.009), and inversely correlated with the relative abundance of both Proteobacteria (*P* = 0.027) and *Propionibacterium* (*P* = 0.015). Alanine was not associated with the number of days elapsed between collection of the urine sample and case onset, and thus appeared to be associated with dysbiosis rather than the host response. Pyridoxine was not as strongly associated with microbial community characteristics, and its association was explained by correlation with alanine (data not shown). Histidine was not independently associated with microbial community characteristics (Table 2), but had a strong inverse association with the number of days between collection of the urine sample and case onset (Spearman’s rho = −0.71, *P* = 0.004). Thus, histidine appeared to be associated with the host response rather than dysbiosis. Because alanine and histidine were observed to have distinct and possibly complementary associations in relation to NEC-I and NEC-II (Table 2 and Figure 5), we examined the ratio of alanine to histidine in relation to NEC outcomes (Figure 5C) and dysbiosis (Table 2). Remarkably, the ratio was positively associated with overall NEC (Kruskal–Wallis, *P* = 0.001), was inversely associated with the relative abundance of *Propionibacterium* (Spearman’s rho = −0.57, *P* = 0.002), and did not differ by NEC sub-type nor in relation to time between sample collection and disease onset.

**Table 2:** **Association between urinary metabolite values**, **microbial community characteristics and NEC**^**a**^

**Characteristic**	**Alanine**	**Histidine**	**Alanine:histidine ratio**
**Spearman**’**s rho** (*P* value)			
Firmicutes, relative abundance	+**0**.**49**	+0.32	+0.20
	(***P*** = **0**.**009**)	(*P* = 0.095)	(*P* = 0.315)
Proteobacteria, relative abundance	−**0**.**42**	−0.24	−0.16
	(***P*** = **0**.**027**)	(*P* = 0.221)	(*P* = 0.413)
*Propionibacterium*, relative abundance	−**0**.**45**	+0.02	−**0**.**57**
	(***P*** = **0**.**015**)	(*P* = 0.920)	(***P*** = **0**.**002**)
**Median values by cluster or NEC status** (Kruskal–Wallis *P* value)			
Cluster I samples versus	**3**.**34**	0.59	**4**.**97**
All other samples	**1**.**52**	0.44	**3**.**46**
	(***P*** < **0**.**001**)	(*P* = 0.140)	(***P*** = **0**.**029**)
Cluster II samples versus	**1**.**52**	0.44	3.51
All other samples	**2**.**46**	0.59	4.86
	(***P*** = **0**.**007**)	(*P* = 0.104)	(*P* = 0.403)
NEC samples versus	2.13	0.33	**6**.**32**
All others excluding non-NEC deaths	1.72	0.53	**3**.**42**
	(*P* = 0.467)	(*P* = 0.169)	(***P*** = **0**.**022**)
NEC-I versus	**2**.**81**	0.55	4.86
All others excluding non-NEC deaths	**1**.**57**	0.44	3.51
	(***P*** = **0**.**023**)	(*P* = 0.394)	(*P* = 0.177)
NEC-II versus	1.50	**0**.**25**	7.35
All others excluding non-NEC deaths	1.84	**0**.**53**	3.53
	(*P* = 0.229)	(***P*** = **0**.**015**)	(*P* = 0.126)

^a^The urine and stool samples were collected from 28 infants between postnatal days 4 to 9. Analyses include 9 NEC cases, 2 deaths and 17 controls, except analysis of NEC, NEC-I and NEC-II, for which the non-NEC deaths were removed as a competing cause. Metabolites measured as normalized peak intensity. Significant values are bolded.

### Predictive biomarkers

To systematically evaluate the signatures identified above as potential predictors of NEC onset, we analyzed the area under the receiver operating curve (ROC), beginning with defined microbial colonization characteristics (Table 3). First, we examined high Firmicutes dysbiosis (≥98% relative abundance in samples from days 4 to 9), which occurred in 4 of 9 NEC cases versus 0 of 18 controls (*P* = 0.007) who had a sample during days 4 to 9. This measure had a good predictive value (72%) and optimal specificity (100%), but very poor sensitivity (44%). Next, we examined the absence of *Propionibacterium* in samples from days 4 to 9 as a potential predictor, which occurred with all NEC cases but only 44% of 18 controls (*P* = 0.009). While the predictive value of the absence of *Propionibacterium* was good (78%) for prediction of NEC, with optimal sensitivity (100%), the specificity was poor (56%). We then examined high Proteobacteria dysbiosis in days 10 to 16 samples as a predictor of subsequent NEC. ROC analysis identified two cut-points for high Proteobacteria dysbiosis, ≥ 90% or ≥98% relative abundance, both of which maximized the predictive value (62%), but neither was significant. We selected the ≥90% cut-point, as it included all NEC-II cases identified in Cluster II/Cluster A by ordination. We then examined the likelihood of developing NEC from having either form of dysbiosis. This analysis, limited to infants with samples in both time periods, found that all 7 NEC cases versus 8 (50%) of 16 controls (*P* = 0.052) had a form of early dysbiosis, which increased the predictive value to 75%. But, the highest predictive value (88%) was obtained from a combination of either form of dysbiosis and lack of *Propionibacterium*, which occurred in 7 of 7 NEC cases and 4 (25%) of 16 controls (*P* = 0.001), and thus, had ideal sensitivity (100%) and good specificity (75%).

**Table 3:** Microbial and metabolite predictors of NEC

**Criteria**	**Description**	**NEC**	**Controls**	***P***^**a**^	**Predictive value *(c)***	**Sensitivity**	**Specificity**
1	High Firmicutes dysbiosis (≥98%, days 4 to 9)	4/9 (44%)	0/18 (0)	0.007	72%	44%	100%
2	No *Propionibacterium* (days 4 to 9)	9/9 (100%)	8/18 (44%)	0.009	78%	100%	56%
3	High Proteobacteria dysbiosis (≥90%, days 10 to 16)	6/9 (67%)	8/19 (42%)	0.420	62%	67%	58%
4	Dysbiosis (criterion 1 or 3)	7/7 (100%)	8/16 (50%)	0.052	75%	100%	50%
5	Combined criteria 2 and 4 (no *Propionibacterium* + dysbiosis)	7/7 (100%)	4/16 (25%)	0.001	88%	100%	75%
6	Urinary alanine:histidine ratio >4 (days 4 to 9)	9/11 (82%)	5/20 (25%)	0.007	78%	82%	75%

^a^*P* values based on Fisher’s exact test. The variations in denominator are due to varying sample availability.

We then examined urinary metabolites as surrogate markers for prediction of NEC. On their own, alanine, pyridoxine and histidine were not significantly associated with a risk of overall NEC. However, when analyzed together, alanine and histidine were significantly associated with NEC (Figure 5C and Table 2). ROC analysis found the optimal cut-point to be a urinary alanine:histidine ratio >4 (Table 3), which yielded a predictive value of 78%. Alanine:histidine ratio values above the cut-point occurred in 9 (82%) of 11 NEC cases and 5 (25%) of 20 controls (*P* = 0.007), providing very good sensitivity (82%) and good specificity (75%).

In summary, the predictive values of variables defining aspects of the early intestinal microbiome or urinary metabolome ranged from 62% to 88%. Each potentially predictive biomarker that we examined was significant except for Proteobacteria dysbiosis, which had the lowest predictive value (62%). Proteobacteria dysbiosis was significant as a predictive variable only if examined together with Firmicutes dysbiosis (75% predictive value). The highest predictive value, 88%, obtained from the combination of either form of dysbiosis and the absence of *Propionibacterium* in the first week of life, suggests the upper limit of prediction that may be obtained from analysis of the early microbial community composition. Given the small sample size, validation is necessary in larger studies as well as other populations. Nevertheless, these data provide strong initial proof of concept that early microbial colonization has a critical role in the development of NEC. This perspective is reinforced by finding a urinary marker (alanine:histidine ratio >4) from analysis of samples from the first week of life, which was associated with microbial community composition and had a predictive value of 78% for prediction of NEC.

## Discussion

Intestinal colonization has long been thought to contribute to NEC in preterm infants, despite a failure to consistently identify a single pathogen or pathogenic microbial community associated with its occurrence. The results of this study indicate the need to focus metagenomic research for preterm infants on the first few weeks of life. Our study revealed several factors that were significantly associated with subsequent risk of NEC, specifically, the absence of *Propionibacterium* in the first week and two distinct forms of dysbiosis that occurred over the first two weeks of life: During days 4 to 9, the microbial community prior to onset of early NEC cases consisted predominantly (≥98%) of Firmicutes, specifically class Bacilli, of which the dominant genera were *Staphylococcus* and *Enterococcus*. This unique Gram-positive microbial signature was not shared by any of the control infants. The second clustered microbial phenotype occurred during 10 to 16 days of life preceding later onset NEC cases, and consisted of a Gram-negative Proteobacteria signature, specifically, family *Enterobacteriaceae*, of which the dominant genera were *Enterobacter* and *Escherichia*. The disparate timing and composition of these high Firmicutes and high Proteobacteria microbial signatures are intriguing, especially given their association with early versus late onset NEC or death. We also found maternal antepartum antibiotic use to be significantly higher in NEC-II compared to NEC-I cases. While there appeared to be a trend towards association of NEC-I with more C-section delivery and NEC-II with more vaginal delivery, this was not significant. Our data also suggested a trend towards lower alpha diversity of samples from later NEC cases compared to controls, but this was also not statistically significant. However, the limited sample size of this study does not preclude the possible differences that might be identified from analysis of larger studies of the early microbiome in future.

While we are not aware of any previous study that has demonstrated two distinct forms of intestinal dysbiosis prior to onset of NEC, previous studies support the plausibility of our findings. Consistent with our finding that distinct forms of dysbiosis were composed of Firmicutes and Proteobacteria, Koenig et al. [51] reported that these phyla are strongly negatively correlated with each other in normal infant colonization. A previous study of 18 preterm infants by Mai et al. [10] reported that the relative abundance of Firmicutes was higher in samples taken one week prior to NEC onset compared to controls, followed by higher abundance of Proteobacteria within 72 hours prior to NEC onset. Although the authors did not describe distinct forms of dysbiosis for distinct subgroups as reported here, their findings nevertheless suggested a key role for both major phyla at distinct timings. Finally, Taur et al. reported two forms of dysbiosis following antibiotic administration in allogeneic hematopoietic transplant patients; consistent with our study, one form of dysbiosis was characterized by dominance of Firmicutes (*Enterococcus* and *Streptococcus*) and the other form was characterized by dominance of various Proteobacteria [57].

Our discovery of two forms of dysbiosis by metagenomic analysis was supported by metabolomics, which identified differences among urine samples that were collected in the first week of life prior to case onset. These two ‘-omic’ methods provide complementary information. Urinary metabolomics is a sensitive method of identifying groups that differ in their intestinal bacterial colonization [25,44]. Production and utilization of specific metabolites differ among colonizing bacteria, which in turn affects their bioavailability to the host [26]. While metagenomic analysis of microbial DNA provides a comprehensive snapshot of bacterial composition, metabolomic comparison of microbial colonization phenotypes provides a snapshot of their differential metabolic activity [27-29]. In our study, three urinary metabolites (alanine, pyridoxine and histidine) differed significantly between one of the NEC sub-types and controls. Urinary alanine was higher in NEC-I compared to controls, positively correlated with the relative abundance of Firmicutes, and negatively correlated with the relative abundance of Proteobacteria and *Propionibacterium*. Alanine is a non-essential amino acid, which is ubiquitously incorporated into bacterial cell wall biosynthesis, a potential target of immune sensing [58-60]. As peptidoglycan constitutes most of the dry weight of Gram-positive organisms but only a small share of the dry weight of Gram-negative organisms, alanine seems a particularly promising candidate to differentiate patients whose microbiome is strongly dominated by Gram-positive organisms. Indeed, alanine was previously reported to be significantly elevated in the feces of irritable bowel syndrome cases compared to controls, and positively associated with higher intestinal colonization with Gram-positive organisms [27]. Pyridoxine, also elevated in the urine of NEC-I infants versus controls, is produced by bacteria in general [61] and may reflect bacterial abundance or growth. But pyridoxine, a correlate of alanine in our data, did not appear to be independently associated with microbial community composition or NEC in the presence of alanine. A proteinogenic amino acid, histidine, differed between NEC-II and controls, but was not associated in our dataset with microbial community composition per se. However, an in vitro study of the metabolites of microbial growth previously reported that histidine was lower in medium with increased growth of *Escherichia*, a genus that contributed to the Proteobacteria dysbiosis associated with NEC-II in our study [25]. While none of the metabolites were alone predictive of overall NEC, the ratio of alanine to histidine was significantly associated with NEC overall as well as with the relative abundance of *Propionibacterium*.

The preterm infants in our study generally lacked microbiota that are known to influence healthy immune development and oral tolerance, including *Bifidobacterium*, *Bacteroides fragilis* and other commensal gut microflora [23,24,62]. While the lack of these beneficial organisms is characteristic of preterm infants, the primers used in this study have been documented to be less than optimal for quantitative representation of *Bifidobacterium*[63]. However, our own unpublished data suggest that the presence or absence of *Bifidobacterium* was unlikely to have been highly biased. In this study, we detected *Bifidobacterium* in 15% of the samples. From the same ongoing cohort of preterm infants, we recently analyzed an additional 182 samples that were amplified using 515 F/806R primers and sequenced on the MiSeq platform [41] and again found a 15% *Bifidobacterium* detection rate. Of the additional samples sequenced both by 515 F/806R (MiSeq) and V3-V5 (454), the same *Bifidobacterium*-positive samples were identified with each method. For one *Bifidobacterium*-*positive* sample, we generated additional data from deep whole genome sequencing (WGS). The estimated relative abundances of *Bifidobacterium* by method were 22% by WGS, 14% by 515 F/806R (MiSeq) and 2% by V3-V5 (454). Taken together, these data suggest that our use of V3-V5 primers did not significantly misrepresent the presence or absence of *Bifidobacterium* colonization in our dataset, but very likely underrepresented the relative abundance of *Bifidobacterium* when present.

*Propionibacterium*, a genus of the phylum Actinobacteria, was the only organism that differed significantly between all NEC cases and controls in this study. The organism was identified in the first postnatal week in about half of the controls but none of later NEC cases, suggesting a potential commensal role. This genus includes many species and strains that are used as probiotics by the dairy industry [54]. Other *Propionibacterium* commonly colonize the skin [64] and have been reported in breast milk [65]. These organisms are so named due to their production of propionic acid as well as other short chain fatty acids that have a beneficial role in intestinal health. The role of *Propionibacterium* in the intestinal colonization of infants is not known. Nevertheless, our observation suggests a benefit from initial colonization with this organism, and the possibility that other commensals may also benefit preterm infants.

The high Firmicutes dysbiosis that we observed may imply excessive exposure to the peptidoglycan that covers the surface of Gram-positive organisms. TLR2 recognizes peptidoglycan, and exhibits excessive signaling in the immature enterocyte [4]. The lack of exposure to LPS-bearing Gram-negative organisms in the first week of life may impair the development of tolerance in preterm infants, resulting in an even higher inflammatory response when presented. The Proteobacteria dysbiosis that we observed was characterized by high abundance of Enterobacteriaceae in the second week of life, consistent with in vivo and ex vivo studies indicating that NEC is a hyperinflammatory state resulting from excessive TLR4 signaling in response to LPS [9-11,13]. However, in our study, high Gram-negative predominance occurred in both NEC and control subjects. High relative abundance of Proteobacteria may induce heightened vulnerability, but the development of NEC may require additional insults or vulnerabilities, including later exposures to pathogens or oxidative stress [66]. Alternatively, control infants with similarly high colonization with Proteobacteria may be more immunologically tolerant. Our finding that no NEC cases but half of the controls had low but detectable levels of *Propionibacterium*, and that the presence of this organism appeared to mitigate the risk of NEC associated with high levels of Proteobacteria in the second week of life, is consistent with the concept that the early presence of commensal bacteria helps induce immune homeostasis [23].

While early colonization might contribute in various ways to the causal pathway leading to NEC, our findings are consistent with the suggestion that early dysbiosis has a time-sensitive role in dysregulation of the developing immune response [19,21]. Supporting this putative role, a time-series analysis of the intestinal transcripts of gnotobiotic and conventionalized mice demonstrated that immune genes respond to microbial colonization in a temporal sequence that coordinates the development of the immune system to achieve homeostasis [20]. For example, T-cell maturation and tolerance-associated functions, such as IL-10 and Foxp3, are significantly increased in conventionalized mice by day eight after introduction of microbiota, a timing relevant to our findings. However, our study does not address causality. Experimental studies are needed of microbial host interactions and immune development of the immature mucosa to understand the potential role of time-specific microbial dysbiosis as related to NEC in preterm infants.

The limitations of this study should also be considered. The sample size of this study was modest, and our findings require validation in larger, multi-site cohorts. Further, we included three non-NEC deaths as a secondary comparison group. The cause of death for the three non-NEC deaths was attributed to respiratory distress syndrome for two infants and suspected infection for one infant. While these deaths may be considered irrelevant to NEC, our data suggest similarity in the microbiome of NEC and the non-NEC (non-congenital) neonatal deaths that occurred in the same postnatal period [7,8,15]. The reason for the observed microbial similarity between high Firmicutes-associated NEC and non-NEC deaths in this study is not known. None of the non-NEC deaths were ever suspected of NEC. The infant with suspected infection had clinical signs of infection and received antibiotics and supportive treatment, but blood cultures were negative. Since the pathobiology of NEC remains an enigma, and no pathogens were identified that may have contributed to the non-NEC deaths, we cannot explain the similarity of the microbiome in non-NEC deaths and early NEC cases. Nevertheless, these initial findings suggest that immune dysregulation secondary to dysbiosis may be common to NEC and some non-NEC deaths.

## Conclusions

Our findings provide important new insights, particularly regarding the role of the early microbiome in subsequent risk of NEC, including early Gram-positive and Gram-negative forms of dysbiosis. Further, the timing of onset of NEC tended to differ by the form of dysbiosis. While older preterm infants tend to have earlier onset of NEC [3], neither infant gestational age nor birth weight explain our findings. All study infants were <29 weeks gestation and <1,200 g birth weight, and neither variable differed between NEC and control infants nor between NEC cases with a microbial phenotype characterized as Firmicutes (Bacilli) or Proteobacteria (Enterobacteriaceae) dysbiosis. We speculate that our findings may be relevant for understanding the variation in timing of NEC onset that has been reported by NICUs [3]. However, future studies are needed of multiple, independent sites to test that hypothesis. Regardless, the discovery of different forms of early microbial dysbiosis provides a focus for investigation of aberrant microbial-mucosal communication as part of the pathobiology leading to NEC.

Our data also indicate that characterization of early dysbiosis and the presence or absence of potentially probiotic organisms, may serve as non-invasive biomarkers, which can together predict NEC in preterm infants. Using the combination of early microbial factors, we obtained a very high predictive value for NEC (88%), but given the small sample size of this study, and its conduct in a single population, this initial estimate should be taken with caution. Rather, the strength of this study is that it provides proof of concept that clinically significant prediction of NEC may be achieved by directly measuring fecal samples, or indirectly through surrogate biomarkers such as urinary metabolites during the first weeks of life. Further characterization of NEC, and potential sub-types of NEC, through –omic approaches and clinical and immunologic assessments of early postnatal life is needed to advance understanding of this complex disease.

### Data availability

The sequence data generated for this work is deposited under the NCBI bioproject ID 63661. Additional sample data can be accessed from the Broad Institute [67]. Metadata is in Additional file 1.

## Abbreviations

1D: One-dimensional; LEfSe: Linear discriminant analysis effect size; LPS: Lipopolysaccharide; NEC: Necrotizing enterocolitis; NICU: Neonatal intensive care unit; NMDS: Non-metric dimensional scaling; NMR: Nuclear magnetic resonance; OTU: Operational taxonomic unit; PCA: Principal component analysis; ROC: Receiver operating curve; rRNA: Ribosomal RNA; TE: Tris-EDTA; WGS: Whole genome sequencing.

## Competing interests

The authors declare no competing interests.

## Authors’ contributions

ALM, KRS, DVW and DSN designed the research. AJL, DHT, and ALM analyzed the data. KRS supervised the clinical research. ALM and DHT supervised sample collection and management. ZY and DHT conducted DNA extractions. DVW and DG guided sequence data production and performed metagenomic analysis. MA performed cluster analysis and guided statistical analyses. MW was responsible for data management. BW and MAK performed the metabolomics research. CH developed analysis tools and guided metagenomic analysis. ALM wrote the paper and all authors contributed to writing or editing the paper. All authors read and approved the final manuscript.

## Supplementary Material

Additional file 1 Metadata; this file provides the clinical data for each infant’s sample, and a data dictionary defining the variables provided. Click here for file

Additional file 2 Supporting Information; this document provides additional data regarding rarefaction; comparison of the clinical factors for the two microbially-defined types of NEC; the extraction protocols and outcomes; and urinary metabolite biomarkers. Click here for file

## References

[B1] Stoll BJ Hansen NI Bell EF Shankaran S Laptook AR Neonatal outcomes of extremely preterm infants from the NICHD Neonatal Research Network Pediatrics 2010 1 443 456 10.1542/peds.2009-2959 20732945 PMC2982806

[B2] Smith PB Ambalavanan N Li L Cotten CM Laughon M Approach to infants born at 22 to 24 weeks' gestation: relationship to outcomes of more-mature infants Pediatrics 2012 1 e1508 e1516 10.1542/peds.2011-2216 22641761 PMC3362905

[B3] Yee WH Soraisham AS Shah VS Aziz K Yoon W Incidence and timing of presentation of necrotizing enterocolitis in preterm infants Pediatrics 2012 1 e298 e304 10.1542/peds.2011-2022 22271701

[B4] Nanthakumar N Meng D Goldstein AM Zhu W Lu L The mechanism of excessive intestinal inflammation in necrotizing enterocolitis: an immature innate immune response PLoS One 2011 1 e17776 10.1371/journal.pone.0017776 21445298 PMC3061868

[B5] Meinzen-Derr J Poindexter B Wrage L Morrow AL Stoll B Role of human milk in extremely low birth weight infants' risk of necrotizing enterocolitis or death J Perinatol 2009 1 57 62 10.1038/jp.2008.117 18716628 PMC2801431

[B6] Quigley M Henderson G Anthony MY McGuire W Formula milk versus donor breast milk for feeding preterm or low birth weight infants Cochrane Database of Systematic Reviews 2007 1 CD002971 CD002971.pub2 10.1002/14651858 17943776

[B7] Cotten CM Taylor S Stoll B Goldberg RN Hansen NI Prolonged duration of initial empirical antibiotic treatment is associated with increased rates of necrotizing enterocolitis and death for extremely low birth weight infants Pediatrics 2009 1 58 66 10.1542/peds.2007-3423 19117861 PMC2760222

[B8] Kuppala VS Meinzen-Derr J Morrow AL Schibler KR Prolonged initial empirical antibiotic treatment is associated with adverse outcomes in premature infants J Pediatr 2011 1 720 725 10.1016/j.jpeds.2011.05.033 21784435 PMC3193552

[B9] Wang Y Hoenig JD Malin KJ Qamar S Petrof EO 16S rRNA gene-based analysis of fecal microbiota from preterm infants with and without necrotizing enterocolitis ISME J 2009 1 944 954 10.1038/ismej.2009.37 19369970 PMC2713796

[B10] Mai V Young CM Ukhanova M Wang X Sun Y Fecal microbiota in premature infants prior to necrotizing enterocolitis PLoS One 2011 1 e20647 10.1371/journal.pone.0020647 21674011 PMC3108958

[B11] Carlisle EM Poroyko V Caplan MS Alverdy JA Liu D Gram negative bacteria are associated with the early stages of necrotizing enterocolitis PLoS One 2011 1 e18084 10.1371/journal.pone.0018084 21445365 PMC3062571

[B12] LaTuga MS Ellis JC Cotton CM Goldberg RN Wynn JL Beyond bacteria: a study of the enteric microbial consortium in extremely low birth weight infants PLoS One 2011 1 e27858 10.1371/journal.pone.0027858 22174751 PMC3234235

[B13] Afrazi A Sodhi CP Richardson W Neal M Good M New insights into the pathogenesis and treatment of necrotizing enterocolitis: toll-like receptors and beyond Pediatr Res 2011 1 183 188 10.1203/PDR.0b013e3182093280 21135755 PMC3125129

[B14] Mihatsch WA Braegger CP Decsi T Kolacek S Lanzinger H Critical systematic review of the level of evidence for routine use of probiotics for reduction of mortality and prevention of necrotizing enterocolitis and sepsis in preterm infants Clin Nutr 2012 1 6 15 10.1016/j.clnu.2011.09.004 21996513

[B15] Morowitz MJ Poroyko V Caplan M Alverdy J Liu DC Redefining the role of intestinal microbes in the pathogenesis of necrotizing enterocolitis Pediatrics 2010 1 777 785 10.1542/peds.2009-3149 20308210

[B16] Parra-Herran CE Pelaez L Sola JE Urbiztondo AK Rodriguez MM Intestinal candidiasis: an uncommon cause of necrotizing enterocolitis (NEC) in neonates Fetal Pediatr Pathol 2010 1 172 180 10.3109/15513811003777342 20450270

[B17] Stuart RL Tan K Mahar JE Kirkwood CD Andrew Ramsden C, et al.: An outbreak of necrotizing enterocolitis associated with norovirus genotype GII.3 Pediatr Infect Dis J 2010 1 644 647 10.1097/INF.0b013e3181d824e1 20589982

[B18] Tai IC Huang YC Lien RI Huang CG Tsao KC Clinical manifestations of a cluster of rotavirus infection in young infants hospitalized in neonatal care units J Microbiol Immunol Infect 2012 1 15 21 10.1016/j.jmii.2011.09.023 22154991

[B19] El Aidy S van Baarlen P Derrien M Lindenbergh-Kortleve DJ Hooiveld G Temporal and spatial interplay of microbiota and intestinal mucosa drive establishment of immune homeostasis in conventionalized mice Mucosal Immunol 2012 1 567 579 10.1038/mi.2012.32 22617837

[B20] Hooper LV Littman DR Macpherson AJ Interactions between the microbiota and the immune system Science 2012 1 1268 1273 10.1126/science.1223490 22674334 PMC4420145

[B21] Martin R Nauta AJ Ben Amor K Knippels LM Knol J Early life: gut microbiota and immune development in infancy Benef Microbes 2010 1 367 382 10.3920/BM2010.0027 21831776

[B22] Reikvam DH Erofeev A Sandvik A Grcic V Jahnsen FL Depletion of murine intestinal microbiota: effects on gut mucosa and epithelial gene expression PLoS One 2011 1 e17996 10.1371/journal.pone.0017996 21445311 PMC3061881

[B23] Kaplan JL Shi HN Walker WA The role of microbes in developmental immunologic programming Pediatr Res 2011 1 465 472 10.1203/PDR.0b013e318217638a 21364495

[B24] Mazmanian SK Liu CH Tzianabos AO Kasper DL An immunomodulatory molecule of symbiotic bacteria directs maturation of the host immune system Cell 2005 1 107 118 10.1016/j.cell.2005.05.007 16009137

[B25] Cevallos-Cevallos JM Danyluk MD Reyes-De-Corcuera JI GC-MS based metabolomics for rapid simultaneous detection of *Escherichia coli* O157:H7, *Salmonella* Typhimurium, *Salmonella* Muenchen, and *Salmonella* Hartford in ground beef and chicken J Food Sci 2011 1 M238 M246 10.1111/j.1750-3841.2011.02132.x 22417363

[B26] Dai ZL Zhang J Wu G Zhu WY Utilization of amino acids by bacteria from the pig small intestine Amino Acids 2010 1 1201 1215 10.1007/s00726-010-0556-9 20300787

[B27] Ponnusamy K Choi JN Kim J Lee SY Lee CH Microbial community and metabolomic comparison of irritable bowel syndrome faeces J Med Microbiol 2011 1 817 827 10.1099/jmm.0.028126-0 21330412 PMC3167923

[B28] Swann JR Tuohy KM Lindfors P Brown DT Gibson GR Variation in antibiotic-induced microbial recolonization impacts on the host metabolic phenotypes of rats J Proteome Res 2011 1 3590 3603 10.1021/pr200243t 21591676

[B29] Tuohy KM Gougoulias C Shen Q Walton G Fava F Studying the human gut microbiota in the trans-omics era – focus on metagenomics and metabonomics Curr Pharm Des 2009 1 1415 1427 10.2174/138161209788168182 19442166

[B30] Walsh MC Kliegman RM Necrotizing enterocolitis: treatment based on staging criteria Pediatr Clin North Am 1986 1 179 201 3081865 10.1016/S0031-3955(16)34975-6 PMC7131118

[B31] Romick-Rosendale LE Brunner HI Bennett MR Mina R Nelson S Identification of urinary metabolites that distinguish membranous lupus nephritis from proliferative lupus nephritis and focal segmental glomerulosclerosis Arthritis Res Ther 2011 1 R199 10.1186/ar3530 22152586 PMC3334650

[B32] Dowd SE Callaway TR Wolcott RD Sun Y McKeehan T Hagevoort RG Edrington TS Evaluation of 16S rDNA-based community profiling for human microbiome research PLoS One 2012 1 e39315 10.1371/journal.pone.0039315 PMC3374619 22720093

[B33] Schloss PD Westcott SL Ryabin T Hall JR Hartmann M Introducing mothur: open-source, platform-independent, community-supported software for describing and comparing microbial communities Appl Environ Microbiol 2009 1 7537 7541 10.1128/AEM.01541-09 19801464 PMC2786419

[B34] Edgar RC Haas BJ Clemente JC Quince C Knight R UCHIME improves sensitivity and speed of chimera detection Bioinformatics 2011 1 2194 2200 10.1093/bioinformatics/btr381 21700674 PMC3150044

[B35] Kostic AD Gevers D Pedamallu CS Michaud M Duke F Genomic analysis identifies association of *Fusobacterium* with colorectal carcinoma Genome Res 2012 1 292 298 10.1101/gr.126573.111 22009990 PMC3266036

[B36] Margulies M Egholm M Altman WE Attiya S Bader JS Genome sequencing in microfabricated high-density picolitre reactors Nature 2005 1 376 380 16056220 10.1038/nature03959 PMC1464427

[B37] Cole JR Wang Q Cardenas E Fish J Chai B The Ribosomal Database Project: improved alignments and new tools for rRNA analysis Nucleic Acids Res 2009 1 D141 D145 10.1093/nar/gkn879 19004872 PMC2686447

[B38] McDonald D Price MN Goodrich J Nawrocki EP DeSantis TZ An improved Greengenes taxonomy with explicit ranks for ecological and evolutionary analyses of bacteria and archaea ISME J 2012 1 610 618 10.1038/ismej.2011.139 22134646 PMC3280142

[B39] Core R Team: R 2012 A Language and Environment for Statistical Computing [http://www.R-project.org]

[B40] Segata N Izard J Waldron L Gevers D Miropolsky L Metagenomic biomarker discovery and explanation Genome Biol 2011 1 R60 10.1186/gb-2011-12-6-r60 21702898 PMC3218848

[B41] Caporaso JG Kuczynski J Stombaugh J Bittinger K Bushman FD QIIME allows analysis of high-throughput community sequencing data Nat Methods 2010 1 335 336 10.1038/nmeth.f.303 20383131 PMC3156573

[B42] Lozupone C Lladser ME Knights D Stombaugh J Knight R UniFrac: an effective distance metric for microbial community comparison ISME J 2011 1 169 172 10.1038/ismej.2010.133 20827291 PMC3105689

[B43] SAS Institute Inc SAS/STAT Statistical Software Version 2011

[B44] Chao A Nonparametric estimation of the number of classes in a population Scand J Stat 1984 1 265 270

[B45] Simpson EH Measurement of diversity Nature 1949 1 688 10.1038/163688a0

[B46] Oksanen J Blanchet FG Kindt R Legendre P O'Hara RB vegan: Community Ecology Package R package version 2011 1 Oksanen J, Blanchet FG, Kindt R, Legendre P, O'Hara RB 17 12 [http://cran.r-project.org/package=vegan]

[B47] McCune B Grace JB Analysis of Ecological Communities 2002 OR, US: Gleneden Beach

[B48] Ward JH Hierarchical grouping to optimize an objective function J Am Stat Assoc 1963 1 236 244 10.1080/01621459.1963.10500845

[B49] Benjamini Y Hochberg Y Controlling the false discovery rate: a practical and powerful approach to multiple testing J Roy Stat Soc Ser B (Methodological) 1995 1 289 300

[B50] Hotelling H The generalization of Student's ratio Ann Math Stat 1931 1 360 378 10.1214/aoms/1177732979

[B51] Koenig JE Spor A Scalfone N Fricker AD Stombaugh J Succession of microbial consortia in the developing infant gut microbiome Proc Natl Acad Sci USA 2011 1 Suppl 1 4578 4585 20668239 10.1073/pnas.1000081107 PMC3063592

[B52] Morowitz MJ Denef VJ Costello EK Thomas BC Poroyko V Strain-resolved community genomic analysis of gut microbial colonization in a premature infant Proc Natl Acad Sci USA 2011 1 1128 1133 10.1073/pnas.1010992108 21191099 PMC3024690

[B53] Yatsunenko T Rey FE Manary MJ Trehan I Dominguez-Bello MG Human gut microbiome viewed across age and geography Nature 2012 1 222 227 22699611 10.1038/nature11053 PMC3376388

[B54] Foligne B Deutsch SM Breton J Cousin FJ Dewulf J Promising immunomodulatory effects of selected strains of dairy propionibacteria as evidenced in vitro and in vivo Appl Environ Microbiol 2010 1 8259 8264 10.1128/AEM.01976-10 20971874 PMC3008228

[B55] McDowell A Barnard E Nagy I Gao A Tomida S An expanded multilocus sequence typing scheme for *Propionibacterium* acnes: investigation of 'pathogenic', 'commensal' and antibiotic resistant strains PLoS One 2012 1 e41480 10.1371/journal.pone.0041480 22859988 PMC3408437

[B56] Kanehisa M Goto S Sato Y Furumichi M Tanabe M KEGG for integration and interpretation of large-scale molecular data sets Nucleic Acids Res 2012 1 D109 D114 10.1093/nar/gkr988 22080510 PMC3245020

[B57] Taur Y Xavier JB Lipuma L Ubeda C Goldberg J Intestinal domination and the risk of bacteremia in patients undergoing allogeneic hematopoietic stem cell transplantation Clin Infect Dis 2012 1 905 914 10.1093/cid/cis580 22718773 PMC3657523

[B58] Duerr CU Hornef MW The mammalian intestinal epithelium as integral player in the establishment and maintenance of host-microbial homeostasis Semin Immunol 2012 1 25 35 10.1016/j.smim.2011.11.002 22138188

[B59] Kurokawa K Gong JH Ryu KH Zheng L Chae JH Biochemical characterization of evasion from peptidoglycan recognition by *Staphylococcus aureus* D-alanylated wall teichoic acid in insect innate immunity Dev Comp Immunol 2011 1 835 839 10.1016/j.dci.2011.03.001 21453720

[B60] Carrasco-Lopez C Rojas-Altuve A Zhang W Hesek D Lee M Crystal structures of bacterial peptidoglycan amidase AmpD and an unprecedented activation mechanism J Biol Chem 2011 1 31714 31722 10.1074/jbc.M111.264366 21775432 PMC3173140

[B61] Tanaka T Kitamoto N Jiang X Estes MK High efficiency cross-reactive monoclonal antibody production by oral immunization with recombinant Norwalk virus-like particles Microbiol Immunol 2006 1 883 888 17116984 10.1111/j.1348-0421.2006.tb03864.x

[B62] Bouladoux N Hall JA Grainger JR Dos Santos LM Kann MG Regulatory role of suppressive motifs from commensal DNA Mucosal Immunol 2012 1 623 634 10.1038/mi.2012.36 22617839 PMC3427718

[B63] Sim K Cox MJ Wopereis H Martin R Knol J Improved detection of bifidobacteria with optimised 16S rRNA-gene based pyrosequencing PLoS One 2012 1 e32543 10.1371/journal.pone.0032543 22470420 PMC3314643

[B64] Blaser MJ Dominguez-Bello MG Contreras M Magris M Hidalgo G Distinct cutaneous bacterial assemblages in a sampling of South American Amerindians and US residents ISME J 2012 1 85 95 22895161 10.1038/ismej.2012.81 PMC3526177

[B65] Jimenez E Delgado S Fernandez L Garcia N Albujar M Assessment of the bacterial diversity of human colostrum and screening of staphylococcal and enterococcal populations for potential virulence factors Res Microbiol 2008 1 595 601 10.1016/j.resmic.2008.09.001 18845249

[B66] Kim M Christley S Alverdy JC Liu D An G Immature oxidative stress management as a unifying principle in the pathogenesis of necrotizing enterocolitis: insights from an agent-based model Surg Infect 2012 1 18 32 10.1089/sur.2011.057 PMC3292758 22217195

[B67] Additional sample data [http://www.broadinstitute.org/annotation/genome/microbiome_projects/InfantGut.html]

