# Supplementary material for: Early microbial and metabolomic signatures predict later onset of necrotizing enterocolitis in preterm infants
Source: Microbiome. 2013 Apr 16;1:13. doi: 10.1186/2049-2618-1-13 (PMC3971624; doi:10.1186/2049-2618-1-13)
Supplement: Additional file 2 — Supporting Information; this document provides additional data regarding rarefaction; comparison of the clinical factors for the two microbially-defined types of NEC; the extraction protocols and outcomes; and urinary metabolite biomarkers. [file 2049-2618-1-13-S2.docx]

**Supporting Information**

Rarefaction. Rarefaction of samples was standardized to 2000 reads for comparisons of alpha and beta diversity. Three samples, all collected during days 4 to 9, had <2000 reads, including one sample from an infant with NEC (specifically, NEC-II) and two samples from controls. These three samples were excluded from analysis of rarefied data. The primary analyses of alpha diversity were based on rarefied data, as the results appeared to differ between rarefied and non-rarefied data. We note, however, that alpha diversity was not significantly associated with later NEC onset in either dataset. For beta diversity, we found the same patterns from the ordination of rarefied (SI, Figure S1) and non-rarefied data (manuscript, Figures 3 and 4). Ordination of either rarefied or non-rarefied data supports the concept of distinct forms and timings of dysbiosis.

Comparison of NEC-I and NEC-II infants. Table S1 compares the clinical characteristics of NEC-I (preceded by high Firmicutes signature in days 4 to 9 of life) and NEC-II (preceded by high Proteobacteria signature in days 10 to 16 of life) cases. T-tests were used to compare continuous variables and Fisher’s exact test was used to compare categorical variables. Only maternal antibiotic use at the time of delivery differed significantly (p<0.05) between NEC-I (no maternal antibiotics out of 4 NEC cases) and NEC-II (maternal antibiotics used by 6 of 7), although primiparity and patent ductus arteriosis trended towards significance (p<0.10). Each variable was then examined in relation for NEC sub-types vs controls. Neither maternal antibiotic use nor PDA differed between the NEC sub-types and controls. However, primiparity was increased in NEC-II (86%) vs controls (24%; p=0.007, Fisher’s exact test) but not NEC-I (24%) vs controls. Because maternal antibiotic use tends to differ by delivery mode, we also conducted an NMDS ordination to visualize the relationship between microbial community, delivery mode and case status during days 4 to 9 and days 10 to 16. Inspection of Figure S2 suggests that vaginal delivery and C-section delivery have distinct microbial communities during days of life 4 to 9 but become indistinguishable by days of life 10 to 16. This trend tends to suggest that NEC-I may be influenced by delivery mode, though the sample size of this study was underpowered to detect differences in microbial communities by NEC type or by delivery mode.

**Table S1. Characteristics of NEC case types**

| **Variable, description** | **NEC type 1**  n= 4 | **NEC type 2**  n=7 |
| --- | --- | --- |
| Birth weight, mean (SD) (g) | 777 (200) | 800 (234) |
| Gestational age, mean (SD) (wk) | 26 (1.7) | 25 (1.7) |
| Non-Hispanic black, no. (%) | 2 (50) | 3 (43) |
| Male, no. (%) | 1 (25) | 5 (71) |
| Patent ductus arteriosus (PDA), no. (%)* | 1 (25) | 6 (86) |
| Primiparous, no. (%)* | 1 (25) | 6 (86) |
| Prenatal steroids given (any), no. (%) | 3 (75) | 7 (100) |
| Hypertension/eclampsia, no. (%) | 1 (25) | 1 (14) |
| Antepartum hemorrhage, no. (%) | 0 (0) | 1 (14) |
| Multiple birth, no. (%) | 1 (25) | 0 (0) |
| Antepartum antibiotic therapy, no. (%)+ | **0 (0)** | **6 (86)** |
| Cesarean section delivery, no. (%) | 4 (100) | 3 (43) |
| Chorioamnionitis, no. (%) | 0 (0) | 1 (14) |
| Antibiotic use in the first wk >5 days, no. (%) | 1 (25) | 3 (43) |

+ Comparison between NEC types significant for maternal antibiotic use, p=0.015, Fisher’s exact

* Difference between NEC types exhibits a statistical trend for primiparity and PDA, p=0.089

Extraction protocol in relation to study findings. Extraction protocol was analyzed for its impact on beta-diversity (Figure S3, Panel A). This involved inspecting the ordination for clustering by extraction protocol. Next, extraction protocol was plotted against each ordination axis individually and again inspected for differences. Then, extraction protocol was used as the outcome variable in a logistic model including each of the three NMDS axes as predictors. None of the axes in the days 4 to 9 or the days 10 to 16 ordinations were significant predictors of extraction protocol, indicating that extraction protocol was not significantly correlated with beta diversity in this study. We then utilized LEfSe to examine taxonomic differences in samples extracted using the Qiagen method vs phenol-chloroform, and found that extraction using Qiagen was associated with significant enrichment for several taxa of bacteria compared to the phenol-chloroform method. However, none of these identified taxa also differed between NEC or NEC sub-type versus controls (Figure S3, Panel B).

We then examined the impact of extraction protocol on alpha-diversity, using extraction protocol as an independent variable in models with Chao1 and Simpson diversity metrics as the dependent variables. Extraction protocol was independently associated (p=0.01) with the Chao1 diversity score but not the Simpson index. However, inclusion of extraction protocol in regression models did not influence the relationship of either diversity metric and NEC, that is, neither Chao1 nor Simpson diversity indexes were significant predictors of NEC after controlling for extraction protocol.

Urinary metabolites. Table S2 provides the results of generalized estimating equation (GEE) models of urine samples collected from days of life 4 to 9. The only exception to that collection window was a case whose only available sample was collected on day of life 3. Inclusion of that sample provided at least one sample from all 11 cases, and 1 or more samples from 20 controls (one control infant lacked any urine sample collection regardless of day of life). Comparisons included all 60 urine samples collected during day of life 4 to 9, but the comparison of NEC-I versus NEC-II and each case sub-type versus controls includes a subset of these 60 samples, as noted in the table. Three metabolites – alanine, pyridoxine, and histidine -- were significantly higher in NEC-I than NEC-II infant urine samples. These metabolites also differed significantly between one of the NEC case sub-types and controls. Two of the metabolites, alanine and pyridoxine, were significantly higher in NEC-I compared to controls. The non-NEC deaths, which, like NEC-I, were preceded by a high *Firmicutes* dysbiosis, also had significantly higher urinary alanine than controls. However, histidine was significantly *lower* in NEC type 2 than controls.

**Table S2**. **Urinary metabolites (samples collected DOL 4 to 9) that differ between NEC types and controls**. Samples were compared using generalized estimating equation (GEE) models. Data are presented as means and standard deviations (SD) for each study group, with p-values indicated for each study group comparison

| **Study group** | No. samples (infants) | **Alanine**  Mean (SD) | **Pyridoxine**  Mean (SD) | **Histidine**  Mean (SD) |
| --- | --- | --- | --- | --- |
| NEC-I | 6 (4) | 3.42 (1.00) | 0.92 (0.28) | 0.60 (0.20) |
| Deaths (non-NEC) | 6 (3) | 2.60 (1.32) | 0.85 (0.19) | 0.61 (0.28) |
| NEC-II | 12 (7) | 1.49 (0.51) | 0.60 (0.17) | 0.33 (0.15) |
| Controls | 36 (20) | 1.73 (0.66) | 0.63 (0.28) | 0.49 (0.19) |
| ***p-values of comparisons*** | | | | |
| NEC-I vs NEC-II | 18 (11) | ***<0.001*** | ***0.003*** | ***<0.001*** |
| NEC-I vs controls | 42 (24) | ***<0.001*** | ***0.040*** | *0.159* |
| NEC-II vs controls | 48 (27) | *0.307* | *0.936* | ***0.013*** |
| Non-NEC deaths vs controls | 42 (23) | ***0.011*** | *0.126* | *0.248* |

**Figure legends**

**Figure S1.** NMDS ordination of OTU level data after rarefaction to 2000 reads, samples from days 4-9 (Panel A) and 10-16 (Panel B). NMDS was based on a weighted UniFrac distance metric, run with 3 dimensions in the vegan package of R. The ordination results resemble those obtained without rarefaction of the data, as shown in the manuscript Figures 3 and 4. The clusters indicated in this ordination were identified by the Ward minimum variance method.

**Figure S2.** NMDS ordination of microbial communities, indicating case-control status and delivery mode. Analysis of microbial communities is indicated for samples from days 4-9 (Panel A) and 10-16 (Panel B). As in previous figures, NMDS was based on a weighted UniFrac distance metric, and run with 3 dimensions in the vegan package of R.

**Figure S3.** Evaluation of extraction protocol. There were 39 samples extracted using the Qiagen protocol and 19 samples extracted using the phenol-chloroform protocol.

**Panel A:** Non-metric multidimensional scaling (NMDS) ordination of OTU level data for samples from infant days of life 4-9 (left panel) and 10-16 (right panel) based on weighted UniFrac distance metric. The black (Qiagen) and open dots (phenol-chloroform) indicate the two different extraction protocols. The boxes along each axis show the spread of samples by extraction protocol on that axis, showing that there are no differences in distribution by extraction protocol. **Panel B:** LEfSe generated linear discriminant analysis (LDA) log scores comparing Qiagen extraction protocol with phenol-chloroform extraction protocol. Taxa that differed between the extraction protocol methods were not considered relevant to the findings of this study.
